# Differences in gut microbiota between allergic rhinitis, atopic dermatitis, and skin urticaria: A pilot study

**DOI:** 10.1097/MD.0000000000025091

**Published:** 2021-03-05

**Authors:** Yu-Jih Su, Sheng-Dean Luo, Chung-Yuan Hsu, Ho-Chang Kuo

**Affiliations:** a Internal Medicine Department; b Department of Otolaryngology; c Pediatrics, Kaohsiung Chang Gung Memorial Hospital and Chang Gung University College of Medicine, Kaohsiung, Taiwan.

**Keywords:** atopic dermatitis, eczema, hives, microbiota, rhinitis, urticaria

## Abstract

**Introduction.:**

Several forms of allergy have been clinically presented, including, among others, atopic dermatitis (eczema), urticaria (hives), and allergic rhinitis (rhinitis). As their detailed pathogenesis continues to be researched, we aimed in the current study to compare gut microbiota differences between eczema, hives, and rhinitis patients.

**Methods.:**

We enrolled 19 eczemas, nine hives, and 11 allergic rhinitis patients in this study. Fecal samples were examined using 16S ribosomal ribonucleic acid amplicon sequencing, followed by bioinformatics and statistical analyses. We compared microbiota in dermatitis (eczema), chronic urticaria (hives), and allergic rhinitis (rhinitis).

**Results.:**

All clinical data were similar between the subgroups. The microbiota results indicated that Bacteroidales species were found in skin allergies, both urticaria and eczema, when compared to rhinitis. The microbiota differs substantially between those patients with atopic dermatitis (eczema), chronic urticaria (hives), and allergic rhinitis (rhinitis), thus indicating that the gut-skin and gut-nose axes exist. Gut flora colonies differ significantly between skin allergy and nose allergy. Bacteroidales species could be a clinical link between gut flora and skin allergy; of those, Bacteroids Plebeius DSM 17135 is significantly associated with the urticaria (hives) subgroup.Conclusion. Our results demonstrated high intra-group homogeneous and high inter-group heterogeneous microbiota. The clinical symptoms of eczema, hives, and rhinitis can all be linked to specific microbiota in the current study. In this pilot study, the Ruminococcaceae and Bacteroidales species are associated with allergic disease, in line with several previous published articles, and the abundance of Firmicutes Phylum is representative of intestinal dysbiosis. In the future, a larger cohort and thorough biochemical studies are needed for confirmation.

## 1 Introduction

Intestinal flora may be associated with various forms of inflammation, including atopic dermatitis,^[1]^ allergy,^[2]^ allergic rhinitis,^[3]^ etc. In this study, we focused on allergic cutaneous disease and allergic rhinitis. The immune reaction between human mucosal immunity, such as in allergic cutaneous disease and allergic rhinitis, and gut micro-organisms has previously been reviewed elsewhere,^[4–6]^ and the gut skin axis^[7]^ has also previously been implicated in the pathogenesis of allergic diseases. Inflammatory skin diseases, such as psoriasis,^[8]^ chronic urticaria (hives),^[9]^ and atopic dermatitis^[1]^ are prevalent nowadays. Although their pathogeneses are still being investigated, the microbiota research on clinical presentation is already available in several articles.^[1,10–12]^ However, few direct comparisons of 3 different diseases’ microbiota have been made in human studies, and only 1 review article^[13]^ was noted.

In this study, we aimed to directly compare microbiota between 3 different clinical presentations of allergic diseases. The benefit of comparing microbiota between these different clinical situations is the potential to clarify the pathogenesis differences and treatment modifications among these allergic diseases. Furthermore, hives research has had relatively small study case numbers; for example, Tao Lu et al enrolled only 20 patients in their study^[14]^; Akram Rezazadeh et al enrolled only 20 cases and 20 controlled patients in their study^[15]^; and Edris Nabizadeh et al also enrolled the same small number of patients in their research.^[9]^ Escherichia coli was higher in chronic urticaria, while Faecalibacterium, Prevotella, and Bacteroides species were all lower in hives.

In atopic dermatitis (eczema), synbiotics has been proven to be effective for certain conditions,^[16]^ thus indicating gut flora's role in this disease. One systemic review article^[12]^ summarized that Staphylococcus species protect against food-related oral sensitization and allergic responses; for example, Clostridia and Firmicutes species relieve milk allergies. However, Lachnospiraceae and Ruminococcaceae species were associated with egg allergy, and Candida and Rhodotorula species lead to atopy. In another allergic skin disease pattern, hives, Lactobacillus, and Bifidobacterium were statistically higher in fecal samples from normal controls compared to patients with hives in a recent study.^[15]^

A common allergic airway disease, allergic rhinitis is associated with allergic asthma.^[17]^ In another study,^[18]^ reduced gut microbiota diversity in infancy was associated with allergic rhinitis and allergy in school age. Asthma may be related to gut microbiota,^[19]^ and several studies^[20,21]^ have indicated that anaerobes species, such as B. fragilis, were associated with asthma. so allergic rhinitis (rhinitis),^[17,22]^ which is a part of allergic disease, may also be related to similar gut flora.^[18]^ While the real mechanism is still under investigation, another researches have mentioned possible mechanisms.^[1,8,17,23,24]^

Although the relationship between these allergic diseases and gut microbiota remains unclear, the direct comparison of fecal samples from these 3 allergic diseases is important for facilitating pathogenesis research and determining lifestyle modifications. The availability of direct comparison data is generally lacking. In the current study, we aimed to compare gut microbiota differences between eczema, hives, and rhinitis patients.

## 2 Methods

### 2.1 Study participants

This study is a prospective controlled observational trial. This study's criteria for atopic dermatitis (eczema), chronic urticaria (hives), and allergic rhinitis (rhinitis) patients consisted of a clinical diagnosis of dermatitis by a senior dermatologist based on his/her clinical judgments obtained before rheumatologists confirm the dermatitis diagnosis after excluding autoimmune disease. Patients with lupus, psoriasis, vasculitis, Sjogren syndrome, or dermatomyositis were excluded.

All patients in the chronic urticaria (hives) subgroup had a history of urticaria confirmed by either a dermatologist or rheumatologist and were regularly followed up at rheumatologist out-patient clinics and used antihistamines to control their symptoms. We excluded the following patients from our study: those with a history of neuropsychiatric diseases, major physical illnesses, diabetes mellitus, neuropathy patients, and patients using probiotics or antibiotics.

Regarding the allergic rhinitis (rhinitis) patients, we enrolled patients who visited the Otorhinolaryngology Department and were confirmed by otorhinolaryngologists to have allergic rhinitis and received regular treatment with either an antihistamine oral medication or nasal spray. None of the patients in this subgroup used antibiotics for treatment.

This study consisted of eligible patients treated in outpatient clinics, with minimum oral medication treatment, at Kaohsiung Chang Gung Memorial Hospital in Taiwan. We obtained written informed consent from the parents or guardians of the participants as required by the Institutional Review Board (IRB) prior to initiating this study. Our research protocol was approved by the IRB at Chang Gung Memorial Hospital in Taiwan (IRB: 201700509B0).

### 2.2 Sample collection

Fecal samples were obtained from atopic dermatitis (eczema), chronic urticaria (hives), and allergic rhinitis (rhinitis) patients using the standard method of placing a piece of feces in a Falcon tube, storing it at 4°C immediately after sample collection, and moving it to a − 80°C refrigerator within 24 h. For the deoxyribonucleic acid (DNA) extraction of the fecal sample, we used a QIAamp DNA Stool Mini Kit (QIAGEN, Tokyo, Japan) according to the manufacturer's instructions. To recover bacterial DNA, samples were pretreated with lytic enzymes prior to extraction using the stool kit. Overall, 100 mg of fecal matter was suspended in 10 mL of Tris– ethylenediaminetetraacetic acid buffer (pH 7.5), and 50 μL of 100 mg/mL lysozyme (type VI) from chicken egg white (MPBIO, Derby, UK) was mixed with 50 μL of 1 mg/mL achromopeptidase (Wako, Osaka, Japan). The solution was incubated at 37°C for 1 hour. Next, 100 μL of 20 mg/mL proteinase K (Wako, Osaka, Japan) was added, followed by incubation at 55°C for 1 hour. The cell lysate was then subjected to ethanol precipitation. The precipitant was dissolved in 1 mL of ASL buffer and subsequently purified using the Stool Mini Kit.

### 2.3 16S ribosomal ribonucleic acid (rRNA) amplicon sequencing

The DNA samples were put into the PCR reaction, where the specified forward primer (TCGTCGGCAGCGTCAGATGTGTATAAGAGACAGCCTCGGGNGGCWGCAG) and reverse primer (GTCTCGTGGGCTCGGAGATGTGTATAAGAGACAGGACTACHVGGGTATCTAATCC) were designed to amplify the V3–V4 genomics region of bacterial 16S rRNA genes. With approximately 550 base pair PCR products confirmed through gel electrophoresis, the products were then sent to library preparation for 16S rRNA sequencing. The DNA library developed pursuant to the 16S rRNA Sequencing Library Preparation instructions (Illumina, California) was made available to us by the manufacturing company. The prepared amplicons were sequenced on the MiSeq sequencer (Illumina, California) using the 600-cycle sequencing reagent and specifying the pair-end mode.

### 2.4 Clinical measurements

Clinical data, including age, leukocyte counts, gender, absolute eosinophil count, platelets, leukocyte differential counts, C-reactive protein (mg/L), erythrocyte sedimentation rate (mm/hour), aspartate transaminase (mg/dL), alanine transaminase (mg/dL), uric acid (mg/dL), creatinine (mg/dL), rheumatoid factor (IU/mL), total cholesterol, and total Immunoglobulin E, were all collected by chart review for atopic dermatitis (eczema), chronic urticaria (hives), and allergic rhinitis (rhinitis) patients.

### 2.5 Statistical and bioinformatics analysis

We analyzed data using the statistical software package Statistical Package for the Social Sciences Software, version 16 (Statistical Package for the Social Sciences Software Inc., Chicago, IL). Variables were presented as either frequency or mean (standard deviation). Two-tailed *P*-values < .05 were considered statistically significant. We adopted the independent Mann–Whitney U test or t-test to determine potential differences in clinical assessments and age between the atopic dermatitis (eczema), chronic urticaria (hives), and allergic rhinitis (rhinitis) patients. The 16S rRNA gene amplicon sequences were analyzed using Mothur v1.39 and the MiSeq SOP. The 16S rRNA V3–V4 sequencing reads were de-multiplexed using MiSeq Reporter v2.6. We assembled the de-multiplexed paired reads into a single contig with the following parameters: minimum length of 405 bp, maximum length of 428 bp, and no ambiguity. The single contig consisted of the effective reads from all samples clustered into operational taxonomic units (OTUs) based on a 97% sequence similarity and then followed sequencing filtering and trimming to de-replicate and align it with the SILVA bacteria reference 16S alignment (132 version) distributed with Mothur (v1.38). We performed sequencing error reduction using PCR chimera removal after screening with UCHIME (4.2 version).

### 2.6 Taxonomy assignment of sequences and clustering into OTU

The Mothur implementation of the Naïve Bayesian Classifier against the homemade RDP rRNA training set (version 9) was created with a taxonomic assignment for every sequence with a minimum bootstrap confidence score of 80%. The clustering of sequences with a threshold identity of 0.03% was carried out using the average neighbor algorithm. Alpha faction analysis, including observed OTUs, Shannon index, observed species index, Chao1, and weighted unifrac Principal Coordinates Analysis plot, were generated using R scripts. We generated the linear discriminant analysis effect size (LEfSe) to determine the taxa most likely to explain the differences between the 3 subgroup samples.

## 3 Results

### 3.1 Demographic data of enrolled patients

Eczema (n = 19), hives (n = 9), and rhinitis (n = 11) patients were enrolled in this study. All clinical data collected by chart review, including age, gender, leukocyte counts, differential counts, absolute eosinophil count, platelets, erythrocyte sedimentation rate (mm/hour), C-reactive protein (mg/L), rheumatoid factor (IU/mL), aspartate transaminase (mg/dL), alanine transaminase (mg/dL), creatinine (mg/dL), uric acid (mg/dL), total Immunoglobulin E, and total cholesterol, did not differ significantly between the eczema (n = 19), hives (n = 9), and rhinitis (n = 11) subgroups (all *P* > .05) (Table 1).

**Table 1:** Demographic data of enrolled patients, in the atopic dermatitis (eczema), hives (U), and allergic rhinitis (AR) subgroups.

	Eczema	*U*	AR	*P* value
Case No.	19	9	11	
Gender	4M/15F	1M/8F	2M/9F	.814
Age	35.7 (28.7,46.6)	34.4 (26.5,46.7)	37 (25.1,39.6)	.701
Leukocytes (/uL)	7.1 (5.72,8.97)	8.1 (7.25,9.3)	6.2 (5.25,8.32)	.265
Neutrophils (%)	58.2 (53.0,67.6)	65.1 (53.6,71.1)	63.1 (56.6,68.6)	.509
Lymphocytes (%)	32.6 (25.2,36.5)	29.2 (18.9,35.1)	28.5 (22.0,34.6)	.490
Monocytes (%)	4.65 (3.67,5.57)	5.3 (3.45,6.85)	5.8 (4.25,8.7)	.432
Absolute eosinophil count	209 (73.2,281.)	203 (109,467)	80.5 (52.5,199.)	.248
Platelets (1000/uL)	269. (247.,317.)	282 (254.,322.)	273 (219,298.)	.753
Hemoglobin (g/dL)	13.6 (12.3,14.5)	13.1 (12.8,14.4)	12.6 (11.9,13.4)	.146
Hematocrit (%)	40.2 (38.0,42.6)	40.1 (38.6,42.6)	37.9 (35.6,40.0)	.150
Alanine transaminase (mg/dL)	18 (11,25)	25 (13.5,43.2)	17 (13.5,31.5)	.692
Creatinine (mg/dL)	0.76 (0.69,1.04)	0.67 (0.44,0.87)	0.62 (0.59,0.72)	.132
Total Immunoglobulin E	122 (18.8,255.)	321. (99.5,3943)	80.5 (24.6,211)	.390
Eosinophil cation protein	14.4 (8.04,28.6)	8.56 (5.7,39)	10.8 (5.72,14.6)	.685
Anti-ENA screen	0.1 (0.1,0.17)	0.1 (0.03,0.2)	0.2 (0.1,0.2)	.239
Erythrocyte sedimentation rate (mm/hour)	14.5 (5.25,37.7)	17 (12,91)	10.5 (7,41)	.649
C-reactive protein (mg/L)	1.85 (0.96,6.22)	9.4 (1.82,16.2)	1.5 (1.23,5.4)	.554

### 3.2 Alpha diversity and beta diversity

**Figure 1:**
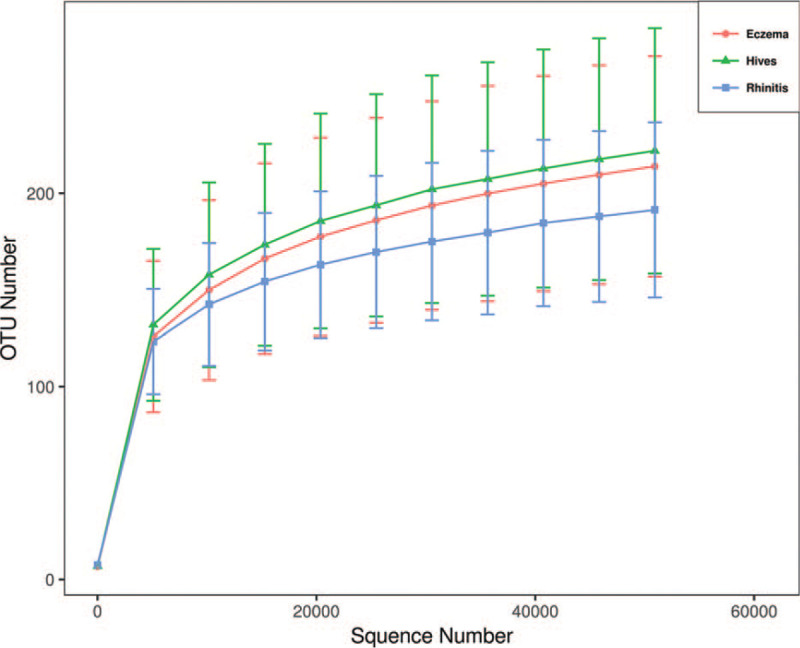
The Rarefaction Curve demonstrates that the atopic dermatitis (eczema), hives (hives), and allergic rhinitis (rhinitis) subgroups were adequately sampled, as well as the species richness of the microbial DNA reach plateau.

**Figure 2:**
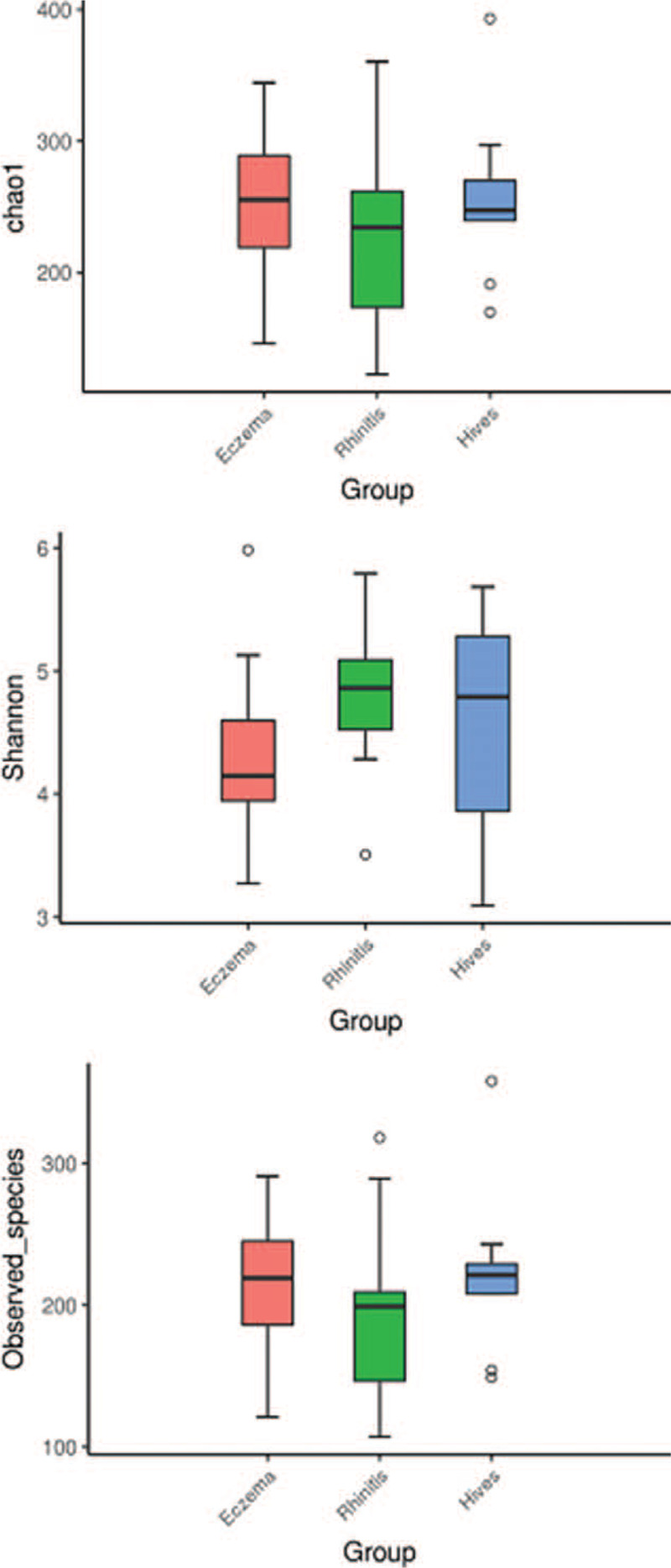
The results of the gut microbiota communities from the atopic dermatitis (eczema), hives (hives), and allergic rhinitis (rhinitis) subgroups show that the Chao index, Shannon index, and observed species index were all *P* > .05, which indicates no distribution differences.

Microbial diversity was assessed either within a subgroup (alpha diversity) or between all 3 subgroups (beta diversity). We calculated several different values to evaluate alpha diversity, including “Rarefaction Curve” to calculate species richness for a given number of an individual subgroup (Fig. 1), “Chao Index” to estimate species abundance (Fig. 2), and “observed species” to approximate the amount of unique OTUs found in each sample (Fig. 2). The Shannon Index (Fig. 2) represents both the abundance and the evenness of the species. As shown in Figure 2, all 3 of these indexes are comparable (*P* > .05).

**Figure 3:**
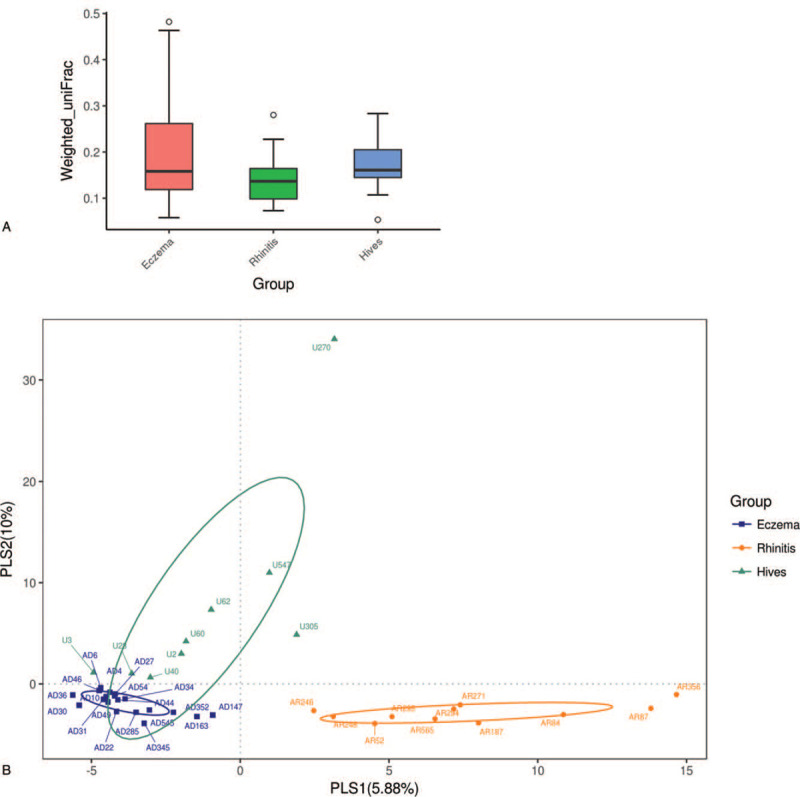
The results demonstrated that the microbial diversity differed significantly in the atopic dermatitis (eczema), hives (hives), and allergic rhinitis (rhinitis) subgroups (Figure 3A). The allergic rhinitis (rhinitis) subgroup was found to significantly differ away from the other 2 subgroups, eczema, and hives (Figure 3B).

The weighted unifrac principal coordinates analysis plot indicates that the gut microbiomes differed between the hives subgroup and the rhinitis subgroup (Fig. 3A, *P* = .046), as well as between the dermatitis subgroup and the rhinitis subgroup (Fig. 3A, *P* = .014). Beta diversity was evaluated using a partial least square discriminated analysis plot to compare all 3 subgroups (Fig. 3B). The results demonstrated that the microbial diversity differed significantly between rhinitis and the other 2 subgroups (Fig. 3B).

### 3.3 Comparing microbiology differences between eczema, hives, and rhinitis subgroups with Welch's t-test, metagenome sequencing, and LEfSe analysis

**Figure 4:**
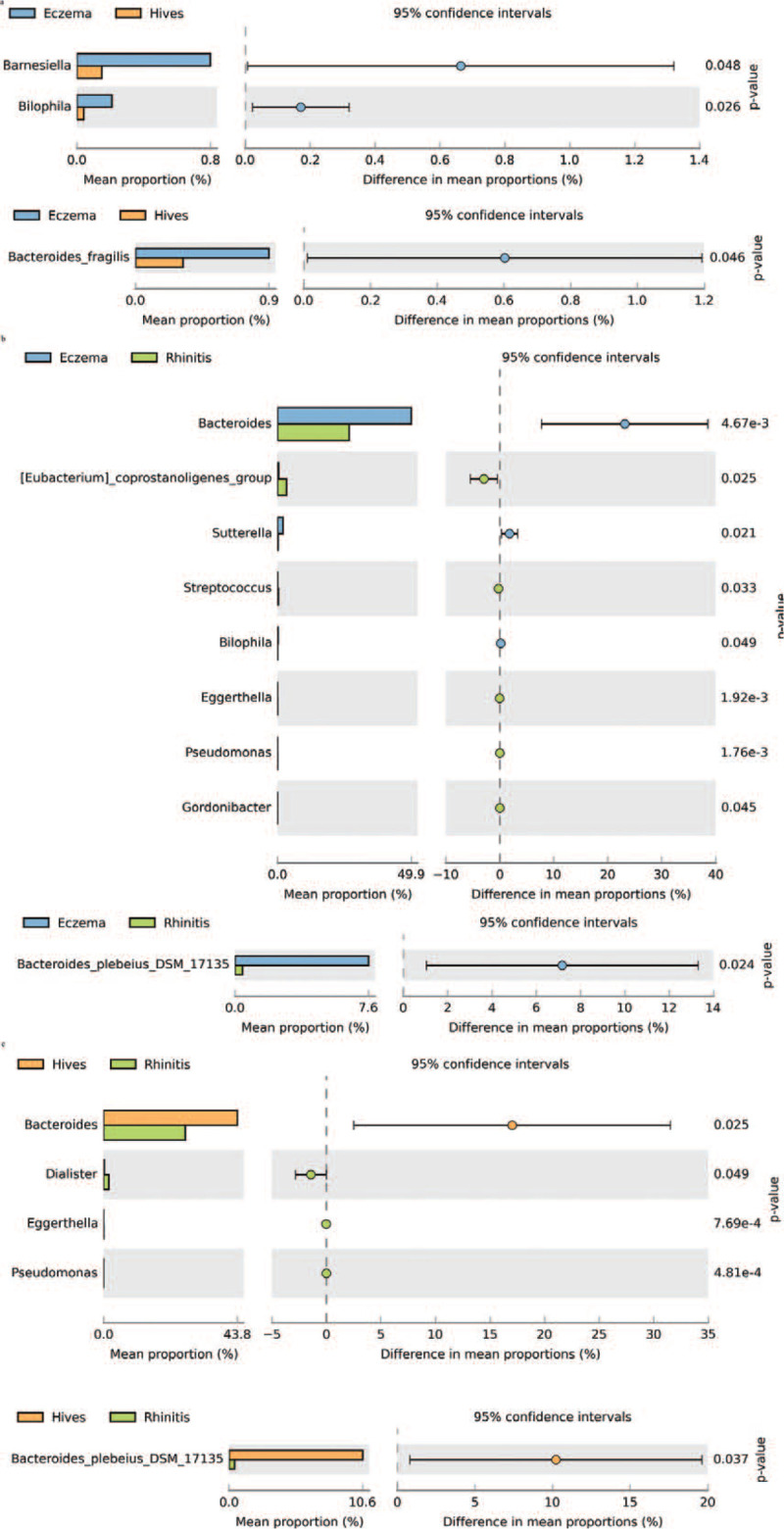
Welch *t*-tests between atopic dermatitis (eczema) and hives (hives) in family and genus (Figure 4A). Welch *t*-tests between atopic dermatitis (eczema) and allergic rhinitis (rhinitis) in family and genus (Figure 4B). Welch *t*-tests between urticaria (hives) and allergic rhinitis (rhinitis) in family and genus (Figure 4C).

We used the Welch's t-test (Fig. 4a) to identify the specific bacteria phylotypes that were differentially altered between eczema (n = 19), hives (n = 9), and rhinitis (n = 11) patients. The Welch test, named for creator B.L. Welch, was adapted from the Student *t*-test^[25]^ to analyze different families of species between the 3 subgroup patients. The results demonstrated that the families Barnesiella and Bilophila, Bacteroids fragilis were significantly higher in eczema than in hives patients (*P* = .048, .026, and .046, respectively, Fig. 4A). Furthermore, the results demonstrated that the families Bacteroides and Sutterella, Bilophila, and Bacteroid plebeius DSM 17135 were significantly higher in eczema than in rhinitis (*P* = .004, .021, .049, and .024, respectively, Fig. 4B). The results also showed that the families Eubacterium coprostanoligenes, Streptococcus, Eggerthella, Pseudomonas, and Gordonibacter were significantly higher in rhinitis than in eczema (*P* = .025, .033, .0019, .0017, and .045, respectively, Fig. 4B). Furthermore, the results indicated that the families Bacteroides, Bacteroid plebeius DSM 17135 were significantly higher in hives than in rhinitis (*P* = .025 and .037, respectively, Fig. 4C). The results also showed that the families Dialister, Eggerthella, and Pseudomonas were significantly higher in rhinitis than in hives (*P* = .049, .0007, and .0004, respectively, Fig. 4B).

**Figure 5:**
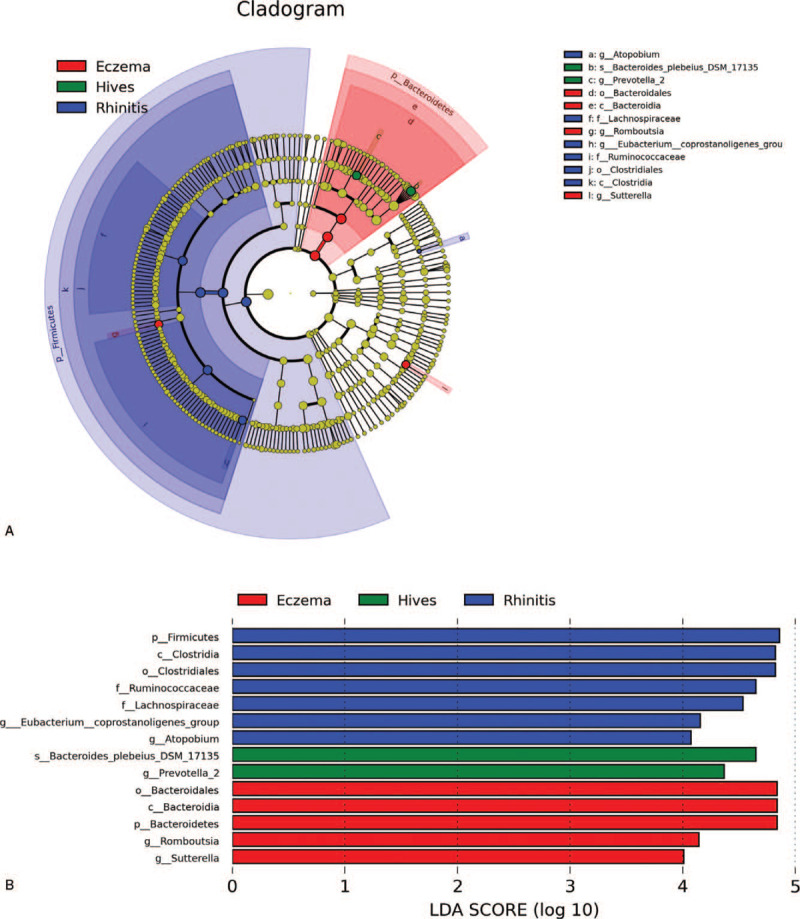
Phylum Firmicutes, class Clostridia, order Clostridiales, families Ruminococcaceae and Lachnospiraceae, genera Eubacterium and atopobium were 10,000 times higher than in the allergic rhinitis (rhinitis) subgroup. Species Bacteroids Plebeius DSM 17135 and genus Prevotella were 10,000 times higher than in the urticaria (hives) subgroup. Order Bacteroidales, class Bacteroidia, phylum Bacteroidetes, and genus Romboutsia were 10,000 times higher than in the atopic dermatitis (eczema) subgroup.

The LEfSe plot of comparing genus and species between eczema (n = 19), hives (n = 9), and rhinitis (n = 11) patients is shown in Figure 5. The relative abundance of order Bacteroidales, Bacteroidia, phylum Bacteroidetes, and genus Romboutsia, Sutterella was significantly higher, 10,000 times, in eczema (n = 19), than in the other 2 subgroups. The relative abundance of Bacteroid plebeius DSM 17135 and Prevotella 2 were significantly higher, 10,000 times, in hives (n = 9) than in the other 2 subgroups. The relative abundance of class Clostridia, order Clostridiales, families Ruminococcaceae, Lachnospiraceae, genera Eubacterium coprostanoligenes, and Atropobium was significantly higher, 10,000 times, in rhinitis (n = 11) than in the other 2 subgroups.

## 4 Discussion

Chronic allergy encompasses several different clinical patterns and organ involvement, including rhinitis,^[26]^ atopic dermatitis,^[27]^ chronic urticaria,^[28]^ conjunctivitis,^[29]^ and asthma,^[30]^ and may even involve oral mucosal,^[31]^ gastrointestinal,^[32]^ and systemic^[33]^ manifestations. Over the past century, treatment has mainly depended on clinical severity, but not specifically on organ involvement, except for cases of asthma.^[34–36]^ As inflammatory cells are homing to different organs, the clinical symptoms are thus initiated. The rationale between the initiation of immune reaction and clinical manifestations of allergy are still emerging,^[19,37]^ and our research has added concrete evidence that gut flora affects clinical target organs involved in allergic diseases. We have shown that the gut flora colonies differ significantly between skin allergy and rhinology allergy, that is, allergic dermatitis and urticaria versus allergic rhinitis (Fig. 3A and 3B).

These days, gut flora is believed to be set by around the age of 2 to 3 years, while bacterial composition is relatively stable throughout each individual's adulthood.^[38]^ Therefore, both the composition and the function of the gut microbiota represent personal related immunological phenotypes, such as atopy, mucosal inflammation, and abdominal gas production from childhood.^[38]^ One systemic review article^[12]^ summarized that Clostridia and Firmicutes species relieve milk allergy, but Lachnospiraceae, Ruminococcaceae species were associated with egg allergy, while Candida and Rhodotorula species led to atopy. In another allergic skin disease pattern, Lactobacillus and Bifidobacterium were statistically lower in patients with hives in a recent study.^[15]^ Whether this phenomenon is associated with disease development has yet to be thoroughly studied, but associative evidence is emerging.^[23,39,40]^ We further indicated that several orders, phyla, and genera were 10,000 times higher in target patient groups indicating significance (see results), with Lachnospiraceae, Ruminococcaceae species being associated with egg allergy already having been mentioned.^[41]^

Allergic skin lesions show infiltration with lymphocytes and macrophages, with the upregulation of leukocyte homing receptors. Clonally expanded cells are noted in atopic dermatitis,^[42]^ which is related to skin flora.^[43,44]^ Furthermore, allergic rhinitis has long been known as a Th2 and partial Th17 immune reaction towards environment allergens.^[45,46]^ The prevalence of Bacteroides in both of the skin allergies in our study, eczema and hives, has been previously connected to the anti-inflammatory effect on food allergies.^[47]^ These results reflect that the allergic pattern may be associated with flora, which may be further associated with gut flora and may also explain our findings in this research.

Regarding patient numbers in this pilot study, we enrolled 39 patients to participate in this research, and the case number was similar to 2 previous chronic urticaria trials: Rezazadeh et al enrolled only 20 cases and 20 healthy controls in their study,^[15]^ and Nabizadeh et al also enrolled 40 patients in their research.^[9]^ Moreover, Tao Lu et al enrolled only 20 subjects (10 patients and 10 controls) in their study,^[14]^ which was only half the size of our study. Therefore, the case number should not be an issue in our study, since our study results are compatible with all the aforementioned previous studies, such as anaerobes were associated with eczema,^[20,21]^ Ruminococcaceae species were associated with egg allergy,^[15]^ and the egg and cow's milk allergy.^[48]^

In summary, we compared the microbiota in eczema, hives, and allergic rhinitis patients. As a result, we linked clinical symptoms of either skin allergy or allergic rhinitis to specific microbiota and made several interesting findings. Gut microbiota links to systemic manifestations of allergic diseases, and the combination of species of gut microbiota could predict clinical symptoms with regard to the allergic site in subjects (Fig. 3). In the current study, the dominant order Bacteroidales species in skin allergies were found in both urticaria and eczema, compared to rhinitis. Furthermore, a previous study already demonstrated the similar result that anaerobes were associated with eczema and asthma.^[21]^ Bacteroids Plebeius DSM 17135 was also significantly associated with the urticaria (hives) subgroup, and we identified several specific Families, Genera, and Species of gut microbiota were associated with either allergic rhinitis, atopic dermatitis, or skin urticaria (Fig. 5). Another interesting finding worth mentioning is that we confirmed that Ruminococcaceae (Fig. 5) was associated with allergic disease as several previous published articles in egg allergy,^[41]^ cow's milk allergy.^[48]^ Among allergic rhinitis patients, the abundance of Firmicutes Phylum (Fig. 5) is representative of intestinal dysbiosis in these patients, which was previously mentioned in another research article.^[49]^ In conclusion, in this pilot study, we have provided several hints that gut microbiota is closely related to allergy symptoms.

## 5 Conclusion

The microbiota differs substantially between those patients with atopic dermatitis (eczema), chronic urticaria (hives), and allergic rhinitis (rhinitis), which implies that the gut-skin and gut-nose axes do exist (Fig. 1).

Gut flora colonies differ significantly between skin allergy and nose allergy, i.e., allergic dermatitis and urticaria versus allergic rhinitis (Fig. 3).

Bacteroidales species could be a clinical link between gut flora and skin allergy; of those, Bacteroids Plebeius DSM 17135 is significantly associated with the urticaria (hives) subgroup (Fig. 4).

In this pilot study, we again confirmed that the anaerobe Ruminococcaceae (Fig. 5) is associated with allergic disease, as has been published in several previous articles, and the abundance of Firmicutes Phylum (Fig. 5) represents intestinal dysbiosis.

## Acknowledgments

We would like to thank the Mitochondrial Research Group at the Chang Gung Memorial Hospital Kaohsiung branch for recommendation.

## Author contributions

CYH, SDL performed the clinical evaluation of patients; HCK participated in the design of the study; YJS drafted the manuscript, conducted the clinical evaluation of patients, sample collection and participated in its design and coordination.

**Conceptualization:** Ho-Chang Kuo.

**Funding acquisition:** Yu-Jih Su.

**Investigation:** Yu-Jih Su.

**Methodology:** Yu-Jih Su, Ho-Chang Kuo.

**Supervision:** Ho-Chang Kuo.

**Validation:** Chung-Yuan Hsu.

**Visualization:** Sheng-Dean Luo.

**Writing – original draft:** Yu-Jih Su.

**Writing – review & editing:** Yu-Jih Su.
